# The Effects of Melatonin Supplementation on Professional Football Player Performance: A Systematic Review

**DOI:** 10.3390/nu15204467

**Published:** 2023-10-21

**Authors:** Antonio Almendros-Ruiz, Alejandro Lopez-Moro, Javier Conde-Pipò, Alfredo Santalla, Bernardo Requena, Miguel Mariscal-Arcas

**Affiliations:** 1 Health Science and Nutrition Research (HSNR-CTS1118), Department of Nutrition and Food Science, School of Pharmacy, University of Granada, 18071 Granada, Spain; aalmendros@correo.ugr.es (A.A.-R.); alexlopez@ugr.es (A.L.-M.); javiercondepipo@gmail.com (J.C.-P.); 2 FSI Lab, Football Science Institute, 18100 Granada, Spain; asanher@upo.es (A.S.); bernardorequena@fsi.training (B.R.); 3 Research Group Physical Activity, Health and Sport CTS-948, Pablo de Olavide University, 41013 Sevilla, Spain; 4 Instituto de Investigación Biosanitaria de Granada (ibs.GRANADA), 18014 Granada, Spain

**Keywords:** football players, melatonin, football performance, soccer, sport nutrition, European football

## Abstract

Background: Melatonin is a hormone that has shown anti-inflammatory actions, reduced oxidative stress, and has effects on physical performance, so the aim of this study was to review the effects of melatonin supplementation on the performance of professional soccer players. Methods: Critical and systematic review. Data were obtained by performing searches in the following bibliographic databases: Web of Science, MEDLINE (via PubMed), Embase, Cochrane Library, and Scopus. The terms used were “Soccer Athlete”, “Melatonin”, and “Soccer Performance”, using “Humans” as a filter. The search update was in May 2023. Results: Having applied the inclusion and exclusion criteria, eight articles were selected out of 59 retrieved references. The dose of melatonin administered in the studies ranged between 5 and 8 mg. The outcomes showed a decrease in oxidative stress, muscle damage, and inflammatory markers in the melatonin-treated group. Conclusions: Exogenously administered melatonin seems to attenuate some of the effects derived from physical exercise, such as oxidative stress, inflammation, and muscle damage, in professional football players, and since it has no potential adverse effects, it could be interesting to apply it in this population. However, the direct effects of melatonin supplementation on physical performance have not been demonstrated, so more research is needed on the intervention period and effective dose and with larger participant populations.

## 1. Introduction

Melatonin is a hormone derived from serotonin and synthesized through the tryptophan–serotonin pathway in the pineal gland, with concentrations varying according to signals from the circadian centers of the brain [1]. This hormone is the current gold standard for evaluating circadian rhythm, as it directly reflects the rhythm in the central nervous system [2]. Melatonin mainly synchronizes the sleep–wake cycle by advancing the oscillatory activity of the major circadian pacemaker and restores the circadian secretion pattern and endogenous levels of melatonin. It is supposed that melatonin administered in the afternoon or early evening, prior to the normal onset of nocturnal melatonin production, can restore disorders related to an altered circadian rhythm [3]. Exogenous melatonin is commonly consumed as a dietary supplement by individuals with difficulty sleeping due to its drowsy effect on the central nervous system [4]. The wide clinical applicability of melatonin supplementation is explained by its relative security, with a low risk of adverse effects [5]. The 5–6 mg dose is the most used in the studies, which appears to be safe and effective and has about a 47 min elimination half-life [6].

In addition, Ishihara et al. [2] add that melatonin plays a crucial role in regulating metabolism and energy balance, as well as lipid and glucose metabolism. It has also been shown that melatonin has anti-inflammatory actions, reduces oxidative stress, and has effects on physical performance [7]. In terms of sporting performance, melatonin supplementation improves exercise recovery after a physical exercise session, which may be linked to decreased muscle oxidative stress induced by melatonin [8]. Kruk et al. [7] detailed some physiological effects that could be related to the increased exercise performance caused by melatonin supplementation, such as increased glucose in muscle, reduced body mass, decreased muscle oxidative stress, prolonged muscle strength, and better adaptation to physical effort. These effects described above could be an ergogenic aid for professional football players, so this systematic review and meta-analysis aimed at verifying the effects of melatonin supplementation on soccer performance.

In the world of professional soccer, there are several factors that affect players’ sleep and rest, such as game schedules, which vary greatly and sometimes occur later than 8:00 p.m., exposure to artificial light, alcohol consumption, or fatigue after travel [9]. All of this creates an environment of sleep deprivation, which can impair recovery processes in soccer players. In addition, as suggested by Fullagar et al. [10], sleep loss can affect physical, neurophysiological, and cognitive parameters, which can have consequences for the performance and recovery of elite athletes.

According to Clemente et al. [11], it has been shown that sleep deprivation inhibits performance in soccer and increases the risk of injury. There are few studies that seek to demonstrate the connection between melatonin and performance in professional soccer. For this reason, the aim of this study was to review and critically analyze the effects of melatonin supplementation on the performance of professional football players, reviewing and summarizing the main findings on the application of melatonin in the performance of professional football players.

## 2. Materials and Methods

Design. Critical analysis of the retrieved studies through systematic technique. The structure of this review followed “the Preferred Reporting Items for Systematic Reviews and Meta-Analyses” (PRISMA) [12] checklist for systematic reviews and was registered in PROSPERO (request code 433832).

Data sources. The data were obtained through an internet search in the following health sciences bibliographic databases: Web of Science (SCIE-WOS), MEDLINE (via PubMed), Embase, Cochrane Library, Scopus, PMC, Embase, PubAg, and AGRIS. The PICO system was used to formulate the questions as follows: P = effect of melatonin on sports performance; I = association of melatonin supplementation with the performance of professional football players; C = melatonin supplementation; and O = changes in the melatonin intake may have a major impact on the performance of professional football players.

Information processing. To define the search terms, the “Thesaurus of Health Sciences Descriptors” (DeCS) developed by the Latin American and Caribbean Centre of Information in Medical Sciences (BIREME) and its equivalence with the “Medical Subject Headings” (MeSH) established by the National Library of Medicine of the United States were consulted.

The following search equations were considered appropriate: soccer players. “Soccer player” [all fields] OR “footballer” [all fields] OR “football player” [all fields] OR “soccer athlete” [all fields] OR “football athlete” [all fields]. “Melatonin” [MeSH Terms] OR “melatonin” [all fields] OR “melatonine” [all fields] OR “melatonin supplement” [all fields]. “Soccer” [MeSH Terms] OR “soccer” [all fields] OR “soccer performance” [all fields] OR “European Football” [all fields] OR “football performance” [all fields] OR “elite football” [all fields] OR “training performance” [all fields] OR “match performance” [all fields].

The final search equation was performed for use in the MEDLINE database via PubMed by joining the three equations previously exposed using Boolean operators Population AND Intervention AND Outcome (following the PIO format), using the “Humans” filter.

Subsequently, this search strategy was adapted to the characteristics of each of the other databases consulted, performing the search from the first available date in each of the selected databases until April 2023. In addition, a manual search was conducted on the references of the articles selected for the review in order to reduce the possibility of publication bias. Risk of bias assessment was performed using the Cochrane blinded risk of bias tool. Articles that fulfilled the following criteria were selected for review and critical analysis: Inclusion: scientific articles that use melatonin supplementation and relate it to performance in football or related sports gestures, articles published in journals indexed in Web of Science (WOS) with JCR, and articles published before April 2023. Exclusion: studies conducted on non-human samples, observational studies that use measures of endogenous melatonin without intervention, and those that study melatonin supplementation in contexts unrelated to football performance or related efforts. Studies published after April 2023 and those whose journals are not included in Web of Science (WOS) without JCR.

Data extraction. To detect duplicate records (those present in more than one database), the ZOTERO program (a reference manager developed by the Center for History and New Media at George Mason University) was used. To systematize and promote understanding of the results, the articles were classified according to the variables under study, considering the following data: first author, year of publication, quality criteria of the journal (quartile and position in the scope area), population studied, country and period of the study, intervention performed, and main results.

Ethical aspects. All data were obtained from articles accepted and published. Thus, in accordance with Law 14/2007 on biomedical research [13], ethics committee approval was not required when using secondary data.

Equity, diversity, and inclusion statement. The authors’ team included senior and junior researchers. This study accounted for the participants’ accessibility needs, regional geographic differences, education, and socioeconomic levels. This study considered gendered and racialized inequities due to the diversity in professional football.

## 3. Results

After applying the search criteria, a total of 59 references were retrieved: 16 (%) in Web of Science (SCIE-WOS), 9 (%) in MEDLINE (via PubMed), 11 (%) in Embase, 12 (%) in Cochrane, and 11 (%) in Scopus, PMC, Embase, PubAg, and AGRIS. The quality of the studies selected on the basis of the inclusion and exclusion criteria were articles indexed in Web of Science with JCR impact factor. The study of the trial’s biases encompassed in the review can be consulted in Table 1.

**Figure 1:**
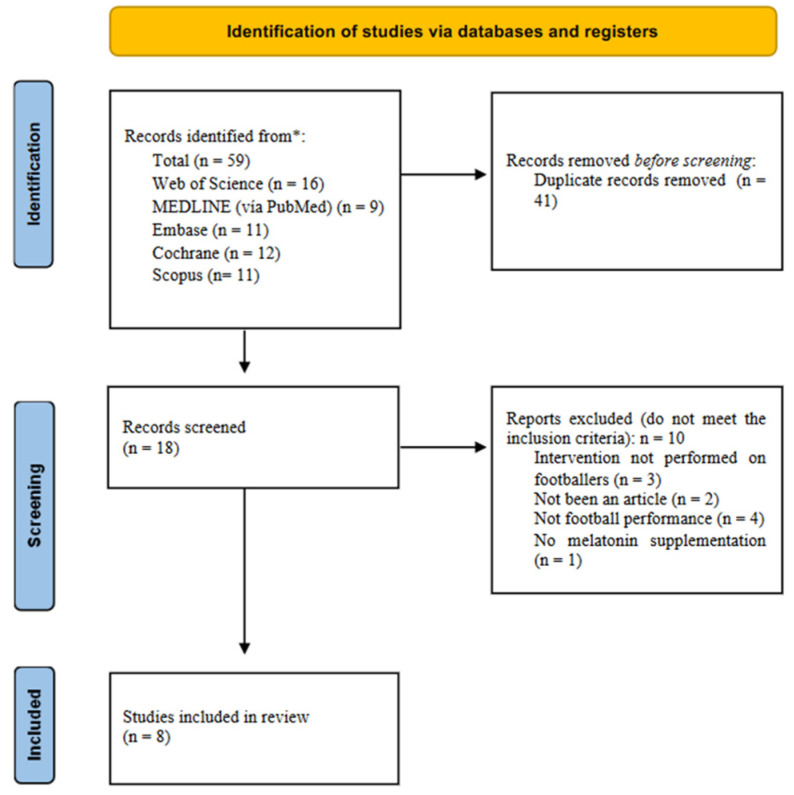
Identification and selection of the studies. * Leading scientific health sciences bibliographic databases.

After removing 41 duplicates, 18 studies were obtained. Based on the inclusion and exclusion criteria and after reading the titles, 10 studies were discarded, and 8 articles were considered appropriate for review and critical analysis, mainly because they did not involve football players as the study population (*n* = 3), they were conference papers rather than articles (*n* = 2), did not involve supplementation with melatonin (*n* = 1), or did not refer to performance in football (*n* = 4) (Figure 1). Table 2 summarizes data from the eight articles included in the review.

According to the selection criteria, all the reviewed articles were experimental studies. Tunisia contributed the highest number of studies, with six articles [15,16,17,19,20,22]. Spain presented one study [18], as did Poland [21]. The study by Czuczejko et al. [21] included the largest number of participants, with *n* = 47, while three studies had the smallest population, with *n* = 12 [15,19,22]. None of the studies included women in their sample. The intervention periods in the reviewed articles ranged from 90 min [18] to 30 days [21].

Performed interventions. The interventions carried out in the reviewed studies reasonably consisted of the administration of exogenous melatonin as supplementation. In most cases, melatonin was ingested between 30 min and 1 h before the physical tests, except for the studies by Czuczejko et al. [21], where it was administered every day 1 h before sleep, and Farjallah et al. 2020 [16], where melatonin was administered every day at 19:00. The doses varied from 5 to 8 mg: four trials used 5 mg of melatonin [16,20,21,22]; three studies administered 6 mg [17,18,19]; and one article was conducted with one group ingesting 5 mg and another group ingesting 8 mg of melatonin [15]. It is noteworthy that none of the studies measured the level of endogenous melatonin before the intervention nor reported monitoring the melatonin ingested through the participants’ diet.

Results of the interventions. In the studies conducted by Ghattasi et al. in 2014 and 2016 [15,22], a reduction in performance was observed after melatonin ingestion. In the 2014 study, the consumption of 8 mg of melatonin prior to nocturnal exercise decreased performance, while in the 2016 study, performance decreased in the morning after melatonin administration but was not affected in the afternoon. In two of the studies conducted by Farjallah et al. [17,19], there were no differences in physical performance parameters between the placebo and melatonin groups. However, in one of these studies, lower inflammation and oral temperature and a decrease in oxidative processes were observed in the melatonin group after exercise [17], while the other study showed reduced liver damage and a protective effect on renal function when melatonin was ingested [19]. Regarding the studies that did show the effects of melatonin administration in football players, five of them demonstrated a decrease in oxidative stress after sports practice when exogenous melatonin was administered [16,17,18,19,21]. Melatonin supplementation also showed anti-inflammatory effects in football players in three of the reviewed studies [16,18,21]. Muscle damage, measured by CK and LDH levels, was attenuated in the group that took melatonin in three studies [16,17,21].

## 4. Discussion

This literature review synthesizes information regarding the effects of exogenous melatonin on the performance of professional football players, providing the scientific community with evidence to promote new interventions for performance enhancement in these athletes. Melatonin is usually used to improve sleep and is normally administered in the afternoon or early evening. The usual dosage to combat sleep problems is 5–6 mg [17,18,19]. Some studies detailed some physiological effects that could be related to the increased exercise performance caused by melatonin supplementation, such as increased glucose in muscle, reduced body mass, decreased muscle oxidative stress, prolonged muscle strength, anti-inflammatory actions, and better adaptation to physical effort [7,20,21,22].

The reviewed articles, excluding Maldonado et al. [18], were published within the last 10 years, and even five out of the eight selected studies were published five years ago, indicating that this is a very current research field with much yet to be investigated [23]. This was evidenced during the literature search conducted in this review, which had to cover a wide time range and generalize the quartiles of the journals in which the articles were published due to the limited publication on this topic. Additionally, there are no studies available on melatonin ingested through food, so a greater number of studies should be conducted to evaluate the intake of melatonin through food and its influence on the endogenous synthesis of this hormone.

Melatonin is rapidly absorbed and reaches peak levels around 40 min [24], so the observed administration schedules (around 30 to 60 min before the tests) would be appropriate.

The intervention period was very short in most studies, with only one study conducted over 30 days. On the one hand, it was demonstrated that melatonin did not improve performance when used with a short-term acute dose [17,19]. On the other hand, it was observed that melatonin is effective as an antioxidant and anti-inflammatory in the short term [16,17,18,19].

Regarding the doses used in the studies, they were heterogeneous and not considered high for the adult population. The standard doses used in the studies ranged from 5 to 8 mg, although more studies are needed to determine the optimal effective minimum dose [25]. However, a Cochrane review [26] stated that a daily dose of melatonin between 0.5 and 5 mg was equally effective, and no better efficacy was attributed to doses higher than 5 mg.

As previously pointed out, the endogenous melatonin of the football players was not taken into account, which poses a problem in validating supplementation. Furthermore, the diets of football players should be controlled and evaluated, as the consumption of certain foods or nutrients such as caffeine or certain vitamins and minerals can alter melatonin production [27], and melatonin has been identified in a wide range of foods, consumption of which can significantly increase melatonin concentration in human serum [28]. Therefore, more studies are needed in which a nutritional assessment of football players is conducted to quantify melatonin consumed through the diet, as the intake of melatonin-rich foods can have positive impacts on health by increasing circulating melatonin [28].

The results of this review demonstrated a decrease in sports performance in football players following the administration of melatonin prior to nighttime exercise, as well as in the case of early morning exercise. In this regard, López-Flores et al. [29] concluded that elevated melatonin concentrations at the time of physical activity could lead to a decrease in sports performance, mainly due to the depressive effects of this hormone on the central nervous system. Additionally, Atkinson et al. [30] suggested that melatonin supplementation had negative effects on reaction time, vigilance, and short-term memory.

The studies did not find direct effects on improving the performance of football players when melatonin was administered. The evidence that melatonin could be an ergogenic aid is weak, and further research is needed before a decision can be made [31].

In contrast, considering the results of the reviewed studies on melatonin and physical performance, it has been observed that melatonin attenuates the decline in physical performance after a period of intensive training rather than enhancing performance itself. This finding can be explained by the improved effect of melatonin on aerobic capacity [32,33].

Another result obtained in the review was the reduction of oxidative stress after physical exercise, which could translate into an indirect improvement in performance. In line with this, numerous studies have demonstrated, both in vitro and in vivo, that melatonin protects against oxidative damage from free radicals [34]. Kruk et al. [7] support these findings by confirming that melatonin and its metabolites play an important role as scavengers of free radicals and ROS/RNS, reducing cellular and tissue oxidative damage.

Several studies included in this review have shown an anti-inflammatory effect due to the ingestion of exogenous melatonin in football players. This result was supported by Ochoa et al. [35], where the obtained data indicated that melatonin administration had protective effects, reducing the activation and overexpression of pro-inflammatory mediators.

Leonardo-Mendonça et al. [36] also found a relationship between melatonin supplementation and the reduction of muscle damage in athletes, as revealed by the results of the present review.

Finally, there is a meta-analysis that demonstrates the effectiveness of melatonin in combating jet lag in athletes who have to undertake international travel as a way to counteract performance impairment [26]. However, this was not the purpose of the present review, although it is proposed as a possible line of future review study due to the importance of jet lag in professional football, given the constant travel requirements of national and international competitions in this sport. The results of this review demonstrated a decrease in sports performance in football players following the administration of melatonin prior to nighttime exercise, as well as in the case of early morning exercise. But in some articles, taken after exercise, it could have some effect on recovery due to the observed antioxidant, muscle damage reduction, and anti-inflammatory effects.

Melatonin intake could be of interest after exercise to improve recovery in professional football players, and because it has no adverse effects, it could be applied without problem, but more studies are needed to draw clear conclusions.

On the other hand, the effects of pre-exercise melatonin supplementation in football players are unclear and could decrease performance; in this aspect, before any intervention, more evidence is needed to establish a protocol.

Limitations. The sample studied is exclusive to professional football, so generalizability to other populations may be limited. It would be necessary to increase the number of studies with a larger number of male and female subjects under study, including football leagues from different countries and continents.

## 5. Conclusions

Melatonin supplementation administration appears to attenuate some of the effects associated with physical exercise, such as oxidative stress, inflammation, and muscle damage in professional football players. Since it does not have potential adverse effects, it could be interesting to apply it to this population. However, the direct effects of melatonin supplementation on physical performance have not been demonstrated, highlighting the need for further research on the intervention period, effective dosage, and larger study populations. Greater emphasis should be placed on conducting studies that assess the nutritional intake of professional football players, as it plays a crucial role in understanding the potential benefits of melatonin in athletes.

## Figures and Tables

**Table 1:** Study of the biases in the trials included in the review [14].

	1	2	3	4	5	6	7
Ghattasi et al., 2014 [15]	No	No	Yes	Yes	No	No	No
Farjallah et al., 2020 [16]	No	No	Yes	Yes	No	No	No
Farjallah et al., 2022 [17]	No	No	Yes	Yes	No	No	No
Maldonado et al., 2011 [18]	No	No	Yes	Unclear	Yes	Yes	No
Farjallah et al., 2022 [19]	No	No	Yes	No	No	Yes	No
Farjallah et al., 2022 [20]	No	No	Yes	Yes	No	No	No
Czuczejko et al., 2019 [21]	No	No	No	Unclear	No	No	No
Ghattasi et al., 2016 [22]	No	No	No	Unclear	Yes	Unclear	No

1. Selection bias (generation of random sequences). 2. Selection bias (concealment of allocation). 3. Performance bias (blinding of participants and staff). 4. Detection bias (blinding of outcome assessment). 5. Attrition bias (unfinished result data). 6. Reporting bias (selective reporting). 7. Other biases (description of other sources of bias).

**Table 2:** Summary of accepted articles for review on the effects of melatonin supplementation on footballers’ performance.

Author, Year	Category	Studied Population	Country	Intervention Period	Intervention Type	Observed Result
Ghattassi et al., 2014 [15]	Category: Biology Rank: 61/85	Professional footballers N: 12 M/F: 12/0 Age: 22.9 ± 1.3 years Height: 1.8 ± 0.05 m Weight: 72.0 ± 8.8 kg	Tunisia	3 nocturnal sessions	Three test sessions were conducted in random order: two sessions with ingestion of melatonin (5 mg or 8 mg) and one session with ingestion of a placebo taken 30 min before the physical test sessions. Performance was measured by the following tests: squat jump (SJ), countermovement jump (CMJ), medicine ball throw (MBT), 5-jump test (5-JT), grip strength (HG), and agility test.	Ingestion of 5 mg of melatonin did not affect subsequent performance. However, the ingestion of 8 mg of melatonin prior to nocturnal exercise decreased performance, specifically in grip strength (HG), compared to placebo (*p* < 0.01) and 5 mg of melatonin (*p* < 0.05). There was a significant decrease when comparing placebo and 5 mg of melatonin with 8 mg of melatonin in squat jump (SJ) and countermovement jump (CMJ) (*p* < 0.01).
Farjallah et al., 2020 [16]	Category: Biology Rank: 34/93	Professional footballers N: 20 M/F: 20/0 Age: 18.81 ± 1.3 years Weight: 70.0 ± 10.6 kg Height: 1.81 ± 0.1 m BMI: 21.27 ± 1.87 kg·m^−2^	Tunisia	7 days	On day 1 and day 7, a blood analysis, an RSA test, and a second blood analysis after the RSA test were conducted. Two training sessions were held each day. One group received a placebo, while the other group received 5 mg of melatonin every day at 7 p.m. in a double-blind fashion.	Melatonin group showed a smaller decrease in performance after the TC (training camp). A decrease in oxidative stress and muscle damage parameters was observed after melatonin ingestion, as well as protection against the decline of antioxidant enzymes. Consumption of melatonin showed an anti-inflammatory effect.
Farjallah et al., 2022 [17]	Category: Sport Sciences Rank: 16/88	Professional footballers N: 13 M/F: 13/0 Age: 17.5 ± 0.8 years Weight: 70.3 ± 3.9 kg Height: 1.8 ± 0.08 m	Tunisia	10 days	On the first day of testing, anthropometric measurements and the Vameval test were conducted. In the second session, one group received 6 mg of melatonin (MEL), and another group received a placebo (PLA) in a double-blind manner. Surveillance tests, oral temperature measurements, and blood samples were taken. Then, the participants performed the maximal incremental test (RET). After exercise, the rating of perceived exertion (RPE) was assessed, and oral temperature and ventilatory thresholds (OT and VT) were measured again. Additionally, a second blood sample was taken 3 min after the RET. In the third session, all tests were repeated under the same conditions so that each participant experienced one session with MEL and another with PLA.	There were no significant differences between MEL and PLA regarding physical performance, heart rate, RPE, and psychocognitive performance. A reduction in oral temperature was observed after exercise in the melatonin group. Regarding biomarkers, there were no significant effects on AOPP or GR after RET. In the PLA group, there was a decrease in GPx, UA, and TBIL levels post-RET and an increase in MDA levels. However, the levels of CK and LDH were lower post-exercise following melatonin ingestion.
Maldonado et al., 2011 [18]	Category: Behavioral Sciences Rank: 19/48	Spanish second-division footballers N: 16 M/F: 16/0 Age: 18–20 years Weight: 68.25 ± 1.53 kg	Spain	90 min	Experimental group (E) was treated with 6 mg of melatonin, while the control group (C) received a placebo. They performed 60 min of intense exercise on a stationary bicycle, during which blood samples were taken under baseline conditions from 9 to 9:30 a.m. Blood samples were also taken 30 min before exercise and at min 3, 15, and 60 during the exercise.	Experimental group showed a lower increase in lipid peroxidation products measured as MDA compared to the control group. In the experimental group, total antioxidant activity did not decrease at 3 and 15 min compared to baseline and increased at 60 min. In the control group, total antioxidant activity decreased at 15–60 min. Experimental group also exhibited significant effects in reducing triglyceride levels and increasing IgA levels after intense exercise.
Farjallah et al., 2022 [19]	Category: Psychology Rank: 59/80	Professional footballers N: 12 M/F: 12/0 Age: 17.54 ± 0.78 years Weight: 70.31 ± 3.86 kg Height: 1.8 ± 0.08 m	Tunisia	3 days	In a randomized, double-blind design, 6 participants took 6 mg of MEL (melatonin), while the other 6 took PLA (placebo). Blood samples were collected 30 min after melatonin ingestion and 3 min after the RET (maximal incremental test). Heart rate, distance covered, time to exhaustion, and various biomarkers were measured.	There were no differences between MEL (melatonin) and PLA (placebo) for physical performance parameters. Ingestion of melatonin showed less liver damage and a protective effect on renal function after exercise.
Farjallah et al., 2022 [20]	Category: Multidisciplinary Sciences Rank: 29/74	Professional footballers N: 20 M/F: 20/0 Age: 18.8 ± 1.3 years Weight: 70.0 ± 10.6 kg Height: 181 ± 8 cm	Tunisia	6 days	Participants’ sleep schedules and meals were monitored and controlled. They underwent two training sessions per day. Two groups were formed: one group took 5 mg of MEL (melatonin), and the other group took PLA (placebo). Blood samples were collected on the first day and after the training period during rest. A battery of physical tests was conducted on the first day and after the training period.	Consumption of MEL (melatonin) reduced exercise-induced oxidative stress. MEL group experienced less muscle, kidney, and liver damage after the intensive training period. The decline in physical performance was attenuated by MEL, which can be explained by the reduction in cellular damage following melatonin supplementation. MEL group exhibited better performance compared to the PLA (placebo) group.
Czuczejko et al. 2019 [21]	Category: Biochemistry & Molecular Biology Rank: 171/297	Football players and control group N: 47 M/F: 47/0 Age: 20.95 ± 2.4 years Weight: 89.7 ± 8.5 kg Height: 1.85 ± 0.2 m	Poland	30 days	Athletes were supplemented with 5 mg of melatonin daily, 1 h before bedtime. Blood samples were collected before starting the supplementation and after. Two physical capacity tests, namely Astrand-Ryhming and PWC170, were conducted.	Supplementation with MEL (melatonin) increased blood level of indolamine in football players. MEL supplementation did not have significant effects on the GSH (antioxidant) concentration. There was a decrease in oxidative processes, indicating a reduction in oxidative stress. The inflammatory markers decreased following the supplementation.
Ghattassi et al., 2016 [22]	Category: Physiology Rank: 73/81	Football players N: 12 M/F: 12/0 Age: 17.9 ± 1.3 years Weight: 62.0 ± 8.8 kg Height: 1.74 ± 0.06 m	Tunisia	2 days	Each participant underwent 2 sessions of physical and cognitive tests with 3 testing periods per day. In one session, they consumed 5 mg of MEL (melatonin) at 07:30 a.m., 30 min before the first test, and in the other session, they took a placebo (PLA). These sessions were separated by a 36 h interval. Participants were advised to maintain a recommended energy intake and adhere to scheduled meal and sleep times.	Ingestion of MEL (melatonin) resulted in a decrease in morning performance in various measures such as vigilance, reaction time, MBT (medicine ball throw), and HG (handgrip strength). There was no significant effect on performance in the afternoon. Interestingly, for both the PLA (placebo) and MEL conditions, performance was better at 4 p.m.

M/F: relationship Male/Female; MEL: melatonin-supplemented group; PLA: placebo group; AOPP: advanced oxidation protein product; GR: reduced glutathione; GPx: glutathione peroxidase; UA: uric acid; TBIL: total bilirubin; MDA: malondialdehyde; CK: creatine kinase; LDH: lactate dehydrogenase.

## Data Availability

There are restrictions on the availability of data for this trial due to the signed consent agreements around data sharing, which only allow access to external researchers for studies following the project’s purposes. Requestors wishing to access the trial data used in this study can make a request to mariscal@ugr.es.
